# Predictive modeling of COPD exacerbation rates using baseline risk factors

**DOI:** 10.1177/17534666221107314

**Published:** 2022-07-09

**Authors:** Dave Singh, John R. Hurst, Fernando J. Martinez, Klaus F. Rabe, Mona Bafadhel, Martin Jenkins, Domingo Salazar, Paul Dorinsky, Patrick Darken

**Affiliations:** Medicines Evaluation Unit, University of Manchester, Manchester University NHS Foundation Hospitals Trust, Manchester M23 9QZ, UK; UCL Respiratory, University College London, London, UK; Joan and Sanford I. Weill Department of Medicine, Weill Cornell Medicine, New York, NY, USA; LungenClinic Grosshansdorf and Christian-Albrechts University Kiel, Airway Research Center North, Member of the German Center for Lung Research (DZL), Grosshansdorf, Germany; Respiratory Medicine Unit, Nuffield Department of Clinical Medicine, University of Oxford, Oxford, UK; AstraZeneca, Cambridge, UK; AstraZeneca, Cambridge, UK; Formerly of AstraZeneca, Durham, NC, USA; AstraZeneca, Gaithersburg, MD, USA

**Keywords:** chronic obstructive pulmonary disease, exacerbations, ICS/LAMA/LABA, machine learning, prediction model, triple therapy

## Abstract

**Background:**

Demographic and disease characteristics have been associated with the risk of chronic obstructive pulmonary disease (COPD) exacerbations. Using previously collected multinational clinical trial data, we developed models that use baseline risk factors to predict an individual’s rate of moderate/severe exacerbations in the next year on various pharmacological treatments for COPD.

**Methods:**

Exacerbation data from 20,054 patients in the ETHOS, KRONOS, TELOS, SOPHOS, and PINNACLE-1, PINNACLE-2, and PINNACLE-4 studies were pooled. Machine learning was used to identify predictors of moderate/severe exacerbation rates. Important factors were selected for generalized linear modeling, further informed by backward variable selection. An independent test set was held back for validation.

**Results:**

Prior exacerbations, eosinophil count, forced expiratory volume in 1 s percent predicted, prior maintenance treatments, reliever medication use, sex, COPD Assessment Test score, smoking status, and region were significant predictors of exacerbation risk, with response to inhaled corticosteroids (ICSs) increasing with higher eosinophil counts, more prior exacerbations, or additional prior treatments. Model fit was similar in the training and test set. Prediction metrics were ~10% better in the full model than in a simplified model based only on eosinophil count, prior exacerbations, and ICS use.

**Conclusion:**

These models predicting rates of moderate/severe exacerbations can be applied to a broad range of patients with COPD in terms of airway obstruction, eosinophil counts, exacerbation history, symptoms, and treatment history. Understanding the relative and absolute risks related to these factors may be useful for clinicians in evaluating the benefit: risk ratio of various treatment decisions for individual patients.

Clinical trials registered with www.clinicaltrials.gov (NCT02465567, NCT02497001, NCT02766608, NCT02727660, NCT01854645, NCT01854658, NCT02343458, NCT03262012, NCT02536508, and NCT01970878)

## Introduction

Exacerbations of chronic obstructive pulmonary disease (COPD) are associated with adverse health outcomes, including greater risk for future exacerbations, lung function decline, worsening quality of life, and increased risk of mortality.^1–3^ In addition, exacerbations account for the majority of healthcare costs associated with COPD, to which those leading to hospitalization contribute significantly.^4^ Accordingly, the prevention of exacerbations is a key goal of COPD management.^5^

Several disease characteristics are known to increase the risk of COPD exacerbations, including previous exacerbation history, greater airflow obstruction or symptom severity, and comorbidities, including diabetes, cancer, heart failure, and gastroesophageal reflux.^6–8^ Also, blood eosinophil count is a predictor of exacerbation risk and a modifier of treatment response to inhaled corticosteroids (ICSs), with greater reductions in exacerbation rates as eosinophil counts increase.^9–13^ Therefore, it is important to tailor interventions according to the individual patient factors that contribute to exacerbation risk.

Current treatment algorithms from the Global Initiative for Chronic Obstructive Lung Disease (GOLD) report recommend using exacerbation history and symptom burden to determine the most appropriate inhaled treatment. Blood eosinophil counts are also considered useful for determining when to use an ICS-containing treatment regimen.^5^ More recently, several predictive models have been developed that incorporate additional clinical and biological characteristics, which may predict future exacerbation risk.^14–17^ Covariates included in these models cover a range of demographic characteristics, previous medication history, and disease severity characteristics such as forced expiratory volume in 1 s percent (FEV_1_%) predicted and exacerbation history; however, the risk of experiencing a COPD exacerbation is also influenced by the effects of pharmacological treatment, which may vary as a function of patient characteristics. Therefore, we used previously collected multinational clinical trial data from more than 20,000 patients to develop a model that would predict the effects of pharmacological treatment on exacerbation risk and apply to individuals within broad populations of patients with COPD.

## Methods

### Source data

The model was developed using data from the Phase III clinical development programs of budesonide/glycopyrrolate/formoterol fumarate metered dose inhaler (BGF MDI), budesonide/formoterol fumarate (BFF) MDI, and glycopyrrolate/formoterol fumarate (GFF) MDI. Patients were randomized to treatment with various combinations of the ICS budesonide (320 or 160 µg), the long-acting muscarinic antagonist (LAMA) glycopyrrolate (18 µg), and the long-acting β_2_-agonist (LABA) formoterol fumarate (FF; 9.6 µg), or placebo, with the specific treatment arms varying by study (Table 1).

**Table 1.** Clinical trial source data by included treatments.

Study	*N*	Study duration	Key inclusion criteria	Treatments included
**ETHOS** ^13^	8509	52 wks	• FEV_1_ 25−<65% • ⩾2 inhaled maintenance therapies • CAT ⩾10 • ⩾1 exacerbation in prior year	BGF 320/18/9.6 µg BGF 160/18/9.6 µg BFF 320/9.6 µg GFF 18/9.6 µg
**KRONOS** ^12^ **+ Extension Studies** ^18,19^	1896	24 wks + 28-wk extensions	• FEV_1_ 25−<80% • ⩾2 inhaled maintenance therapies • CAT ⩾10 • No exacerbation requirement	BGF 320/18/9.6 µg BFF 320/9.6 µg (MDI) BFF 320/9 µg (DPI) GFF 18/9.6 µg
**TELOS** ^20^	2361	24 wks	• FEV_1_ <80% • ⩾1 inhaled maintenance therapy • CAT ⩾10 • No exacerbation requirement	BFF 320/9.6 µg (MDI) BFF 320/9 µg (DPI) BFF 160/9.6 µg BD 320 µg FF 9.6 µg
**SOPHOS** ^21^	1843	12–52 wks (variable)	• FEV_1_ 25−<80% • ⩾1 inhaled maintenance therapy • CAT ⩾10 • ⩾1 exacerbation in prior year	BFF 320/9.6 µg BFF 160/9.6 µg FF 9.6 µg
**PINNACLE-1** ^22^ **+ PINNACLE-3 Extension Study** ^23^	2096	24 wks + 28-wk extension	• FEV_1_ <80% • No requirements for inhaled maintenance therapy, symptoms, or exacerbations	GFF 18/9.6 µg GP 18 µg FF 9.6 µg Placebo^a^
**PINNACLE-2** ^22^ **+ PINNACLE-3 Extension Study** ^23^	1609	24 wks + 28-wk extension	• FEV_1_ <80% • No requirements for inhaled maintenance therapy, symptoms, or exacerbations	GFF 18/9.6 µg GP 18 µg FF 9.6 µg Placebo^a^
**PINNACLE-4** ^24^	1740	24 wks	• FEV_1_ <80% • No requirements for inhaled maintenance therapy, symptoms, or exacerbations	GFF 18/9.6 µg GP 18 µg FF 9.6 µg Placebo

BD, budesonide; BFF, budesonide/formoterol fumarate; BGF, budesonide/glycopyrrolate/formoterol fumarate; CAT, COPD Assessment Test; COPD, chronic obstructive pulmonary disease; DPI, dry powder inhaler; FEV_1_, forced expiratory volume in 1 s; FF, formoterol fumarate; GFF, glycopyrrolate/formoterol fumarate; GP, glycopyrrolate; MDI, metered dose inhaler; wk(s), week(s).

*N* represents the modified intent-to-treat populations in ETHOS, KRONOS, TELOS, and SOPHOS, and the intent-to-treat populations in PINNACLE-1, PINNACLE-2, PINNACLE-3, and PINNACLE-4.

a Patients in the placebo arm were not eligible to continue into the extension study.

The database included pooled exacerbation data from a total of 20,054 patients from ETHOS (NCT02465567),^13^ KRONOS (NCT02497001),^12^ TELOS (NCT02766608),^20^ SOPHOS (NCT02727660),^21^ PINNACLE-1 (NCT01854645),^22^ PINNACLE-2 (NCT01854658),^22^ and PINNACLE-4 (NCT02343458)^24^ (Table 1). Data from extension studies (up to 1 year in duration) of KRONOS (NCT03262012, NCT02536508)^18,19^ and PINNACLE-1 and PINNACLE-2 (PINNACLE-3; NCT01970878)^23^ were also included. All treatments were delivered *via* a single Aerosphere inhaler (AstraZeneca), except for the BFF dry powder inhaler (Symbicort Turbuhaler; AstraZeneca) used in KRONOS and TELOS.

All studies enrolled patients 40–80 years of age with moderate-to-very severe COPD [FEV_1_/forced vital capacity (FVC) ratio < 0.7 and FEV_1_ of  < 80% predicted (<65% in ETHOS)] and a smoking history of ⩾10 pack-years. In addition, SOPHOS and ETHOS required a history of ⩾1 exacerbation in the previous year. The PINNACLE studies did not have any entry criteria regarding prior treatment or symptoms; all other studies required that patients were symptomatic [COPD Assessment Test (CAT) score ⩾10] despite receiving ⩾1 (TELOS, SOPHOS) or ⩾2 (KRONOS, ETHOS) COPD maintenance medications at study entry.

### Model development

The endpoint of interest was the annualized rate of moderate/severe exacerbations (defining moderate exacerbations as those that require treatment with systemic corticosteroids or antibiotics, or both, and severe exacerbations as those that require hospitalization or those that resulted in death). Exacerbation data only included events that occurred during randomized treatment. Modeling was conducted using the statistical software R, and both machine learning techniques and traditional statistical modeling approaches were utilized.

A preliminary model was developed using negative binomial generalized linear modeling (GLM) with data from all studies except ETHOS. A statistical analysis plan was finalized, including steps that would be completed following the unblinding of ETHOS data. Predictors were investigated based on prior literature reporting clinical, physiological, and demographic risk factors for exacerbations.^8,9^ The set of proposed predictors included blood eosinophil count (log-transformed), ICS use, sex, FEV_1_ (post-bronchodilator percent predicted), exacerbation history (number in last year), smoking status (current/former), CAT score, prior maintenance therapies, and average daily reliever medication use (in puffs/day). Interaction terms with budesonide were proposed for ICS use, eosinophil count, smoking status, and eosinophil count by smoking status.

Following the completion of the ETHOS study, a wide range of prospectively named potential predictors available in all studies were considered for the final model development. These additional potential predictors included age, body mass index, height, race, duration of COPD, GOLD classifications A–D, prior ICS use, prior LAMA use, prior LABA use, number of pack-years smoked, number of severe exacerbations in the last year, blood neutrophil count, medical history of gastroesophageal reflux disease, cardiometabolic medical history (including diabetes, hypertension, and high cholesterol), region, study, percent reversibility to salbutamol, and other lung function parameters such as FVC, forced expiratory flow at 25–75% of FVC (FEF_25–75_), and peak expiratory flow (PEF). No patients had missing exacerbation outcomes, and no covariate had greater than 1.25% missing data. As such, only complete cases were used in model development.

The pooled dataset was randomly split into a training set and a test set (stratified by study and treatment), containing 85% and 15% of the population, respectively, to develop the final model (following unblinding of the ETHOS data). Among each pair of covariates with a correlation ⩾0.75, one predictor was chosen based on clinical relevance and precedent, leaving a set of predictors taken forward to machine learning. Machine learning methods – including gradient boosting^25^ (with virtual twins),^26,27^ GLMtree,^28–30^ GUIDE,^31,32^ and Elastic Nets^33^ – were used on the training set to assess variable importance, confirm proposed predictors, and identify additional predictors, including interactions with treatment terms, which would add predictive value. Additional predictors of interest were then incorporated into the final negative binomial GLM. Time at risk was used as an offset variable. This selection was further informed by backward variable selection to ensure the model was parsimonious, retaining covariates or interactions with *p* < 0.1, or up to *p* = 0.2 if there was considerable prior literature supporting their inclusion. Treatment covariates were included to ensure unconstrained prediction was possible for each combination therapy.

In addition to the full model, a simplified model was also tested, including only three predictors known to be available in most patient care settings (exacerbation history, eosinophil count, and prior ICS treatment). Results for the full and simplified models were compared to determine the value of the additional predictors.

Model fit was assessed on the training and test sets using rootograms to compare the predicted distribution of the number of exacerbations with the observed distribution at the population level. Model fit was also assessed on the test set using the median absolute difference between observed and predicted exacerbation rates, and for the prediction of patients with 0 *versus* ⩾1 exacerbation in the following year, in terms of area under the receiver operating characteristic (ROC) curve, positive predictive value, and negative predictive value.

From the final models, rate ratios (RR) and 95% confidence intervals (CIs) were used to present each predictor’s role. Predicted exacerbation rates for a selection of example patients were derived, setting other covariates to typical values close to the median or mode for the dataset.

## Results

### Population characteristics

Overall, 19,194 patients had complete data available and were included in the model development. The population included patients from North America, South America, Europe, Asia, South Africa, and Australasia.

The demographic and disease characteristics of the training set (*n* = 16,314) and test set (*n* = 2880) are shown in Table 2. Demographics were comparable between the two datasets. A majority of patients in both datasets (92%) had moderate or severe COPD, and 65% had experienced ⩾1 moderate or severe exacerbation in the past year. The mean CAT score was approximately 19 in both datasets (range: 0–40).

**Table 2.** Population characteristics of the training and test sets.

	Training set (*n* = 16,314)	Test set (*n* = 2880)
Age, years		
Mean (SD)	64.3 (7.8)	64.6 (7.9)
Range	40–81	40–80
Male sex	10,022 (61.4%)	1734 (60.2%)
Race		
White	13,270 (81.3%)	2332 (81.0%)
Asian	1865 (11.4%)	335 (11.6%)
Black	692 (4.2%)	131 (4.5%)
Other	487 (3.0%)	82 (2.8%)
Region		
United States and Canada	8066 (49.4%)	1429 (49.6%)
Western Europe	2318 (14.2%)	428 (14.9%)
Eastern Europe	2038 (12.5%)	371 (12.9%)
Latin America	1551 (9.5%)	235 (8.2%)
China	1205 (7.4%)	202 (7.0%)
Asia (non-China)	584 (3.6%)	119 (4.1%)
Australasia and South Africa	552 (3.4%)	96 (3.3%)
Smoking status		
Current smoker	7245 (44.4%)	1319 (45.8%)
Former smoker	9069 (55.6%)	1561 (54.2%)
Mean COPD duration, years (SD)	7.8 (6.2)	7.7 (6.1)
Disease severity		
Mild	28 (0.2%)	3 (0.1%)
Moderate	7066 (43.3%)	1250 (43.4%)
Severe	7986 (49.0%)	1397 (48.5%)
Very severe	1232 (7.6%)	230 (8.0%)
FEV_1_% predicted		
Mean (SD)	48.3 (13.1)	48.1 (13.2)
Range	19–95	16–88
Mean reliever medication use, puffs/day (SD)	3.1 (3.3)	3.2 (3.3)
Exacerbation history in the past year		
⩾1 moderate/severe	10,646 (65.3%)	1868 (64.9%)
⩾1 severe	2258 (13.8%)	393 (13.6%)
Blood eosinophil count		
Geometric mean, cells/mm^3^	162	160
<100 cells/mm^3^	2824 (17.3%)	52 (18.8%)
100–<300 cells/mm^3^	10,776 (66.1%)	1847 (64.1%)
⩾300 cells/mm^3^	2714 (16.6%)	491 (17.0%)
CAT score		
Mean (SD)	19.1 (6.8)	19.2 (6.7)
Range	0–40	0–40
Treatment received		
BGF 320/18/9.6 µg	2278 (14.0%)	402 (13.9%)
BGF 160/18/9.6 µg	1782 (10.9%)	314 (10.9%)
BFF 320/9.6 µg^a^	3545 (21.7%)	625 (21.7%)
BFF 160/9.6 µg	1050 (6.4%)	186 (6.5%)
GFF 18/9.6 µg	3585 (22.0%)	633 (22.0%)
BD 320 µg	168 (1.0%)	30 (1.0%)
GP 18 µg	1148 (7.0%)	202 (7.0%)
FF 9.6 µg	2190 (13.4%)	387 (13.4%)
Placebo	568 (3.5%)	101 (3.5%)
Prior maintenance treatment		
ICS/LAMA/LABA	4795 (29.3%)	807 (28.0%)
ICS/LABA	5318 (32.6%)	958 (33.3%)
LAMA/LABA	2287 (14.0%)	428 (14.9%)
ICS/LAMA	245 (1.5%)	36 (1.3%)
ICS only	364 (2.2%)	54 (1.9%)
LAMA only	935 (5.7%)	152 (5.3%)
LABA only	332 (2.0%)	69 (2.4%)
None	2038 (12.5%)	376 (13.1%)

BD, budesonide; BFF, budesonide/formoterol fumarate; BGF, budesonide/glycopyrrolate/formoterol fumarate; CAT, COPD Assessment Test; COPD, chronic obstructive pulmonary disease; DPI, dry powder inhaler; FEV_1_, forced expiratory volume in 1 s; FF, formoterol fumarate; GFF, glycopyrrolate/formoterol fumarate; GP, glycopyrrolate; ICS, inhaled corticosteroid; LABA, long-acting β_2_-agonist; LAMA, long-acting muscarinic antagonist; MDI, metered dose inhaler; SD, standard deviation.

Data are *n* (%) unless otherwise specified.

a Includes BFF MDI (320/9.6 µg) and DPI (320/9 µg).

### Model development

Signal searching was carried out to determine optimal predictors. Results of important prognostic predictors from gradient boosting are shown in Figure S1 in the Online Supplement (other machine learning results not shown). The expected model covariates (based on prior literature) of exacerbation history, COPD severity (by FEV_1_% predicted), eosinophil count, symptoms (by CAT score), prior therapies, and sex were all confirmed as important.

Region was added to the final full model, and prior maintenance therapies were incorporated using separate factors for prior ICS use, prior LAMA use, and prior LABA use to provide a complete characterization of prior treatment history. Smoking status was not found to be of high importance but was retained due to knowledge from the literature and its potential to be important in interaction terms.^9^ Several additional spirometry parameters (e.g. FEF_25–75_, reversibility, and PEF) were found to be important, but given their correlation with FEV_1_% predicted or limited availability in clinical practice, they were not added to the model. The predictor variable relating to study (ETHOS, KRONOS, etc.) was removed from the model to increase generalizability.

Based on results from machine learning, several variables were determined to potentially show a differential response depending on the use of budesonide-containing therapy in the following year. As a result, expected interaction terms with eosinophil count, prior ICS use, and smoking status were retained. Additional interactions with exacerbation history, prior LABA use, and reliever medication usage were included, as well as an interaction between eosinophil count and smoking status. A three-way interaction between budesonide use, eosinophil count, and smoking status was not found to be of value, as the relationship between eosinophil count and the benefit of budesonide did not vary significantly depending upon smoking status. The backward selection step also removed interactions between budesonide use and FEV_1_% predicted, and between glycopyrrolate use and exacerbation history.

In the final full model, a higher number of exacerbations in the prior year, higher eosinophil count, each additional prior maintenance treatment (ICS, LAMA, or LABA), a higher number of puffs/day of reliever medication, lower FEV_1_% predicted, female sex, higher CAT score, region, and current smoking were found to be significant predictors of exacerbation risk, with prior exacerbations, eosinophil count, and prior therapy as modifiers of ICS response (Table 3). Full model coefficients for the final model are provided in Table S1 in the Online Supplement.

**Table 3.** Predictive performance of full and simplified prediction models of the rate of moderate or severe COPD exacerbations.

	Full model		Simplified model	
Model covariates	Moderate/severe COPD exacerbation rate ~ offset[Log (Exposure)] + Intercept		Moderate/severe COPD exacerbation rate ~ offset[Log (Exposure)] + Intercept	
Prognostic covariates	+ No. of exacerbations in prior year*** + log(Eosinophils)*** + Prior ICS use*** + Prior LABA use*** + Prior LAMA use*** + Mean daily reliever medication usage*** + FEV_1_% predicted*** + Sex*** + CAT score*** + Region*** + Smoking status** + log(Eosinophils):Smoking status**		+ No. of exacerbations in prior year*** + log(Eosinophils)*** + Prior ICS use***	
Treatment covariates	BD*** + GP*** + FF + BD:FF + GP:FF^#^ + BD:GP:FF		BD*** + GP^#^ + FF** + BD:FF + GP:FF^†^ + BD:GP:FF	
Interactions with ICS treatment	+ BD × No. of exacerbations in prior year* + BD × log(Eosinophils)*** + BD × Prior ICS use^#^ + BD × Prior LABA use* + BD × Mean daily reliever medication usage^†^ + BD × Smoking status		+ BD × No. of exacerbations in prior year*** + BD × log(Eosinophils)*** + BD × Prior ICS use***	
Performance metrics	Training set	Test set	Training set	Test set
Median difference between predicted and actual rate	0.77	0.77	0.86	0.87
Area under ROC curve for prediction of 0 *versus* ⩾1 exacerbations	0.70	0.71	0.67	0.65
Positive predictive value	47%	48%	44%	45%
Negative predictive value	80%	80%	80%	80%

ANOVA, analysis of variance; BD, budesonide; CAT, COPD Assessment Test; COPD, chronic obstructive pulmonary disease; FEV_1_, forced expiratory volume in 1 s; FF, formoterol fumarate; GP, glycopyrrolate; ICS, inhaled corticosteroid; LABA, long-acting β_2_-agonist; LAMA, long-acting muscarinic antagonist; ROC, receiver operating characteristic.

Negative binomial generalized linear models.

Significance of sequential inclusion in model from ANOVA: ***p < 0.001; **p < 0.01; *p < 0.05; ^†^*p* < 0.10; ^#^*p* < 0.20.

Model fit, as assessed using rootograms, demonstrated that the distribution of the predicted number of exacerbations in the following year was similar to the actual distribution with a median absolute difference between actual and predicted exacerbation rates of 0.77 for the full model. The area under the ROC curves, at 0.70, demonstrated reasonable prediction of patients with and without an exacerbation in the following year, and performance metrics were similar in both the training set and test set (see Figure S2 in the Online Supplement). For a negative predictive value of 80%, the training and test sets showed positive predictive values of 47% and 48%, respectively, for the full model (Table 3).

Prediction metrics were ~10% better, in relative terms, in the full model than in the simplified model, based only on exacerbation history, eosinophil count, and ICS use (Table 3; Table S2 in the Online Supplement). The relationship between eosinophil count and exacerbation rates was similar in the full and simplified models.

### Prediction of exacerbation rates

**Figure 1.**
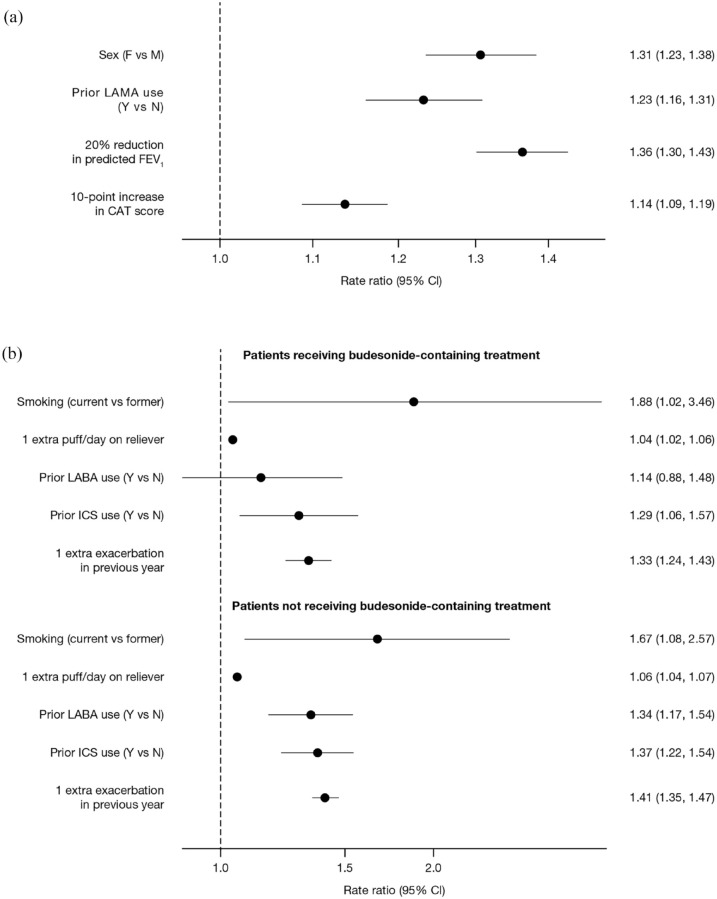
Predictive factors of annual moderate/severe exacerbation rates: (a) main effects and (b) interaction terms with budesonide. CAT, COPD Assessment Test; CI, confidence interval; COPD, chronic obstructive pulmonary disease; F, female; FEV_1_, forced expiratory volume in 1 s; ICS, inhaled corticosteroid; LABA, long-acting β_2_-agonist; LAMA, long-acting muscarinic antagonist; M, male; N, no; Y, yes.

The impact of selected prognostic factors on exacerbation rates, regardless of treatment in the following year, is illustrated in Figure 1.

The following main effects were associated with increased risk of an exacerbation, but were not found to modify the relative benefit of any of the treatments: female sex (RR: 1.31, 95% CI: 1.23–1.38); prior LAMA use (RR: 1.23, 95% CI: 1.16–1.31); FEV_1_% predicted (RR: 1.36, 95% CI: 1.30–1.43 for a 20% reduction in FEV_1_% predicted); and CAT score (RR: 1.14, 95% CI: 1.09–1.19 for a 10-point increase in CAT score) (Figure 1(a)).

Current smoking, a higher number of puffs/day of reliever medication, prior LABA use, prior ICS use, and additional COPD exacerbations in the previous year were associated with increased risk of a moderate/severe exacerbation, with a differential response depending on budesonide use (Figure 1(b)).

**Figure 2.**
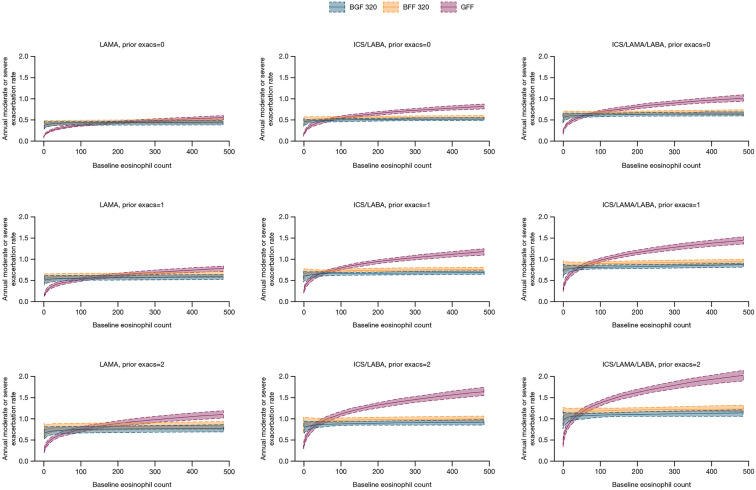
Predicted annual moderate/severe exacerbation rate by blood eosinophil count (cells/mm^3^) according to prior therapy and exacerbation history. BFF, budesonide/formoterol fumarate; BGF, budesonide/glycopyrrolate/formoterol fumarate; CAT, COPD Assessment Test; COPD, chronic obstructive pulmonary disease; exacs, moderate/severe exacerbations; FEV_1_, forced expiratory volume in 1 s; GFF, glycopyrrolate/formoterol fumarate; ICS, inhaled corticosteroid; LABA, long-acting β_2_-agonist; LAMA, long-acting muscarinic antagonist. Banded areas denote the standard error. For all panels, results are for a patient with COPD with the following characteristics: 65-year-old, former smoker, from North America, FEV_1_ 45% of predicted, CAT score of 20, using three puffs/day of reliever. For the training set, each panel represents the following proportion of patients in the source population: LAMA only, 0 exacs = 3.8%; LAMA only, 1 exacs = 1.3%; LAMA only, 2 exacs = 0.5%; ICS/LABA, 0 exacs = 9.7%; ICS/LABA, 1 exacs = 11.7%; ICS/LABA, 2 exacs = 9.4%; ICS/LAMA/LABA, 0 exacs = 4.5%; ICS/LAMA/LABA, 1 exacs = 12.0%; ICS/LAMA/LABA, 2 exacs = 9.9%.

The model was then applied to several example patient types to illustrate the predicted exacerbation rate with various treatments, according to blood eosinophil count, prior therapy, and exacerbation history. Results are shown in Figure 2 for a patient with the following characteristics, representing the approximate median values for the dataset: former smoker, from North America, FEV_1_ 45% of predicted, CAT score of 20, and using three puffs/day of reliever medication. Consistent with KRONOS and ETHOS results,^12,13^ these predictions show a greater benefit of ICS-containing treatments over LAMA/LABA treatment in patients with higher eosinophil counts (regardless of prior treatment and exacerbation history), with the magnitude of expected benefit increasing as eosinophil counts increased.

Larger benefits were also demonstrated in patients with more prior maintenance therapies and a greater number of previous exacerbations.

## Discussion

We developed models predicting moderate/severe exacerbation rates that could be applied to patients with COPD with a broad range of clinical and physiological features, including airway obstruction, blood eosinophil counts, exacerbation history, symptoms, and treatment history. These models allow for the comparison of various COPD treatments and an examination of their relative efficacy in different subgroups of patients, highlighting those who may derive the greatest benefit from triple therapy or ICS-containing therapies. Highly significant predictors included exacerbation history, FEV_1_% predicted, eosinophil count, sex, region, CAT score, prior treatment, and reliever medication use. These risk factors may be used to judge the potential benefits of switching between treatments for a broad range of patients with COPD, not only those who require step-up due to continued symptoms or exacerbations.

Given that patients experience an integer number of exacerbations in a year, but predicted rates are continuous, the full model showed good agreement between predicted and observed exacerbations rates, with a positive predictive value of 48%, for a negative predictive value of 80%. Metrics were provided for a high negative predictive value such that patients were not falsely predicted to have no exacerbations. False-positive predictions of exacerbations in the following year would also occur, as illustrated by the positive predictive value. However, in clinical terms, false positives were considered less of a concern than false negatives and are inevitable when predicting a transient outcome (even patients with established exacerbation risk may not experience one every year).

Even a single exacerbation can result in negative health outcomes for patients.^1^ Therefore, proactively identifying patients predicted to have a high rate of exacerbations and optimizing treatment to prevent future exacerbations should be a key aim of COPD management. Notably, many of the risk factors shown to be important in our model can be modified or improved (e.g. FEV_1_% predicted, smoking status, and CAT score), suggesting that exacerbation risk can be modulated through treatment and lifestyle changes. In addition, while current GOLD recommendations do incorporate symptom burden, exacerbation history, and eosinophil count as key factors in treatment decisions,^5^ our model quantifies the potential absolute differences in predicted exacerbation rates based on these parameters in patients receiving various treatments. These absolute differences may be more informative than relative risk reductions for healthcare providers to evaluate the benefit:risk ratio of various treatment decisions for individual patients. For example, a smaller relative treatment benefit may substantially impact patients with a high expected rate of exacerbations. In contrast, a larger relative benefit may have a more limited impact in those with a low expected exacerbation rate. The prediction of absolute exacerbation rates may also be useful when planning clinical trials to assess the likelihood of COPD exacerbations in different patient groups. For trials that require the occurrence of exacerbations to provide useful data, predicted rates could be used to enrich trial populations for patients most at risk of exacerbations.

As expected, our model showed that the greatest treatment benefits of ICS-containing treatments *versus* a LAMA/LABA would be predicted in patients with prior ICS use, prior exacerbation history, and a high eosinophil count. However, benefits of ICSs were observed even in patients without a history of exacerbations in the past year (particularly among those with high eosinophil counts). The reason for this observation may be that, in patients with prior ICS use, a lack of exacerbations in the previous year suggests that these patients had a positive response to their ICS treatment. While taking into account the limitations of documenting only 1 year of exacerbation history, these findings suggest that the use of ICSs, even in patients without recent exacerbations, may help prevent their occurrence in the future. Given that a single COPD exacerbation is associated with lung function decline and other adverse health outcomes,^1^ predicting the first event, and not just future events, is important for those without a history of previous exacerbation.

In general, our models agree with previous reports of risk factors for COPD exacerbations that were based on randomized controlled trials^9–11,34^ or observational studies.^6–8^ In line with the findings from these studies, prior exacerbation history and the severity of airflow obstruction and symptoms were among the most significant predictors of exacerbation rates in our models. However, in contrast to the findings of Bafadhel *et al.*,^9^ the impact of smoking status was less substantial in our study. We did not find that the relationship between exacerbations, eosinophil count, and budesonide use varied significantly according to smoking status, although there were interactions for budesonide use by smoking status and budesonide use by eosinophil count. The reasons for this are unclear but may relate to the populations studied. In addition, while observational studies have found that comorbidities were strong predictors of future exacerbations,^6–8^ the clinical trials used to develop our models had exclusion criteria for clinically significant, uncontrolled diseases other than COPD, limiting the presence of some common comorbidities in our source data.

While several other predictive models for COPD exacerbations have been published,^14–17,35–37^ our models have several strengths compared with previous work. Many of the previously published predictive models for COPD exacerbations used source data from a single country or region.^16,17,35–37^ In contrast, our model was derived from a broad patient population, including patients from all populated continents with a wide range of prior inhaled treatments (from short-acting bronchodilators only to ICS/LAMA/LABA) and exacerbation histories in the prior year (0 to >2). The geographically comprehensive range of regions that were included, encompassing different standards of care and diversity in patient behavior and characteristics, should improve the model’s generalizability.

Furthermore, to the best of our knowledge, our models are the first to predict absolute exacerbation rates for patients on various pharmacological treatments. While most predictive models for COPD exacerbations report relative risks according to various patient factors, the ACCEPT model also predicted absolute rates for different patient characteristics.^15^ However, it is difficult to compare performance metrics between these models as they are influenced by the follow-up duration of the source clinical trials and the prevalence of exacerbations in the population. Notably, in contrast to the current work, while the ACCEPT source population had longer follow-up on average, it did not include any patients without prior exacerbations in the previous year, exacerbations rates were not predicted according to possible future pharmacological treatments, and the role of eosinophils was not considered, which, as we have shown, is essential in predicting response to ICS-containing therapy. To the best of our knowledge, this is the first description of the application of machine learning to the prediction of exacerbation rates in patients with COPD. Although previous studies have used machine learning techniques to assess COPD-related problems,^38,39^ previously published predictive models of exacerbation risk in COPD have not utilized machine learning.^14–17,35–37^

Several limitations of our study population should also be noted. None of the clinical trials used to develop the models included patients with mild airflow obstruction, patients with a concurrent asthma diagnosis, or never-smokers. Thus, the model cannot be considered reliable for these patient groups. Furthermore, although the overall patient population was broad, some therapies were assessed primarily in patients with low risk (e.g. monotherapy) or high risk (e.g. triple therapy) of exacerbations (see Table 1). Therefore, the modeling relied on the assumption that the relative benefits of different treatments follow similar patterns across the span of included patients. The source trials also included only one drug from each class (ICS, budesonide; LAMA, glycopyrrolate; LABA, FF) and have not yet been demonstrated to be generalizable across all drugs in these classes. While clinical trial data provided reliable and unbiased information on treatment response and a wide selection of potential predictors, there may be differences in relative treatment benefits in clinical trials *versus* real-world clinical practice. Future studies are needed to validate our models during real-world use and determine whether predictions are generalizable at the drug class level, in order to optimize their utility in clinical practice.

Some of the prognostic factors included may not be regularly available in clinical practice, particularly in primary care, limiting the practical applicability of the full model. For this reason, a simplified model was developed with only three predictors (exacerbation history, eosinophil count, and prior ICS use). Performance metrics were approximately 10% greater in the full model than the simplified model (area under the ROC curve 0.71 *versus* 0.65; median absolute difference 0.77 *versus* 0.87). The simplified model may be particularly useful in primary care situations or when up-to-date spirometry and CAT score assessments are unavailable. However, we also aimed for the full model to be parsimonious, recognizing the risk of overfitting and the effort involved to utilize a large number of risk factors. Therefore, not all predictors that were identified in machine learning were included in the final model. In general, those that were not included tended to consistently appear relatively low in priority order compared with the factors that were included in the model or, alternatively, were highly related to factors that were included.

In conclusion, we developed two models to predict exacerbation rates for patients with COPD receiving treatment with various combinations of ICS, LAMA, and LABA. These models illustrate the various risk factors that should be considered when judging the exacerbation risk of individual patients with COPD, and may help inform treatment decision-making, selection of clinical trial populations, and assessment of population-level health risks.

## Supplemental Material

sj-docx-1-tar-10.1177_17534666221107314 – Supplemental material for Predictive modeling of COPD exacerbation rates using baseline risk factors Click here for additional data file. Supplemental material, sj-docx-1-tar-10.1177_17534666221107314 for Predictive modeling of COPD exacerbation rates using baseline risk factors by Dave Singh, John R. Hurst, Fernando J. Martinez, Klaus F. Rabe, Mona Bafadhel, Martin Jenkins, Domingo Salazar, Paul Dorinsky and Patrick Darken in Therapeutic Advances in Respiratory Disease

sj-xlsx-2-tar-10.1177_17534666221107314 – Supplemental material for Predictive modeling of COPD exacerbation rates using baseline risk factors Click here for additional data file. Supplemental material, sj-xlsx-2-tar-10.1177_17534666221107314 for Predictive modeling of COPD exacerbation rates using baseline risk factors by Dave Singh, John R. Hurst, Fernando J. Martinez, Klaus F. Rabe, Mona Bafadhel, Martin Jenkins, Domingo Salazar, Paul Dorinsky and Patrick Darken in Therapeutic Advances in Respiratory Disease
