# Supplementary material for: Predictive modeling of COPD exacerbation rates using baseline risk factors
Source: Ther Adv Respir Dis. 2022 Jul 9;16:17534666221107314. doi: 10.1177/17534666221107314 (PMC9340368; doi:10.1177/17534666221107314)
Supplement: sj-docx-1-tar-10.1177_17534666221107314 – Supplemental material for Predictive modeling of COPD exacerbation rates using baseline risk factors [file sj-docx-1-tar-10.1177_17534666221107314.docx]

Online Supplement 1

Table S1. Full model coefficients.

|  | Estimate | Std. error | *z*-value | *p*-value | Sig. |
| --- | --- | --- | --- | --- | --- |
| *For all patients* | | | | | |
| (Intercept) | –1.054 | 0.232 | –4.54 | <0.001 | ^***^ |
| Region – Australasia and South Africa | 0.403 | 0.065 | 6.24 | <0.001 | ^***^ |
| Region – Asia (non-China) | 0.028 | 0.091 | 0.31 | 0.761 |  |
| Region – China | 0.092 | 0.063 | 1.46 | 0.145 | ^#^ |
| Region – Eastern Europe | –0.280 | 0.049 | –5.67 | <0.001 | ^***^ |
| Region – Latin America | –0.081 | 0.048 | –1.70 | 0.089 | ^†^ |
| Region – Western Europe | 0.126 | 0.041 | 3.08 | 0.002 | ^**^ |
| No. of exacerbations in prior year | 0.344 | 0.023 | 15.25 | <0.001 | ^***^ |
| log(Eosinophils) | 0.120 | 0.038 | 3.19 | 0.001 | ^**^ |
| FEV_1_ % predicted | –0.015 | 0.001 | –12.84 | <0.001 | ^***^ |
| Prior ICS use | 0.315 | 0.060 | 5.28 | <0.001 | ^***^ |
| Prior LABA use | 0.294 | 0.069 | 4.25 | <0.001 | ^***^ |
| Prior LAMA use | 0.208 | 0.031 | 6.77 | <0.001 | ^***^ |
| Mean daily reliever medication usage (puffs/day) | 0.055 | 0.006 | 8.76 | <0.001 | ^***^ |
| Sex – female | 0.267 | 0.029 | –9.22 | <0.001 | ^***^ |
| CAT score | 0.013 | 0.002 | 5.75 | <0.001 | ^***^ |
| Smoking status: current smoker | 0.511 | 0.220 | –2.32 | 0.020 | ^*^ |
| Treated with BD 160 µg | 0.709 | 0.337 | 2.10 | 0.036 | ^*^ |
| Treated with BD 320 µg | 1.321 | 0.319 | 4.14 | <0.001 | ^***^ |
| Treated with GP | –0.238 | 0.117 | –2.04 | 0.041 | ^*^ |
| Treated with FF | –0.039 | 0.105 | –0.37 | 0.710 |  |
| Treated with GP and FF | 0.062 | 0.129 | 0.48 | 0.632 |  |
| Treated with BD 320 µg and FF | –0.121 | 0.211 | –0.58 | 0.566 |  |
| Treated with BD 160 µg, GP and FF | 0.071 | 0.095 | 0.75 | 0.453 |  |
| Treated with BD 320 µg, GP and FF | 0.087 | 0.070 | 1.25 | 0.211 |  |
| log(Eosinophils): Smoking status – current smoker | –0.127 | 0.042 | –3.03 | 0.002 | ^**^ |
| *Interaction terms for those receiving budesonide only* | | | | | |
| BD 160 µg: No. of exacerbations in prior year | –0.044 | 0.039 | –1.13 | 0.258 |  |
| BD 320 µg: No. of exacerbations in prior year | –0.058 | 0.031 | –1.88 | 0.061 | ^†^ |
| BD 160 µg: log(Eosinophils) | –0.114 | 0.060 | –1.89 | 0.058 | ^†^ |
| BD 320 µg: log(Eosinophils) | –0.220 | 0.046 | –4.79 | <0.001 | ^***^ |
| BD 160 µg: Prior ICS use | –0.031 | 0.100 | –0.31 | 0.755 |  |
| BD 320 µg: Prior ICS use | –0.061 | 0.079 | –0.77 | 0.442 |  |
| BD 160 µg: Prior LABA use | –0.398 | 0.140 | –2.85 | 0.004 | ^**^ |
| BD 320 µg: Prior LABA use | –0.162 | 0.114 | –1.42 | 0.155 | ^#^ |
| BD 160 µg: Smoking status – current smoker | 0.054 | 0.077 | 0.69 | 0.488 |  |
| BD 320 µg: Smoking status – current smoker | 0.121 | 0.062 | 1.93 | 0.053 | ^†^ |
| BD 160 µg: Mean daily reliever medication usage | 0.009 | 0.011 | 0.79 | 0.432 |  |
| BD 320 µg: Mean daily reliever medication usage | –0.017 | 0.009 | –1.92 | 0.055 | ^†^ |

ANOVA, analysis of variance; BD, budesonide; CAT, COPD Assessment Test; COPD, chronic obstructive pulmonary disease; FEV_1_, forced expiratory volume in 1 second; FF, formoterol fumarate; GP, glycopyrrolate; ICS, inhaled corticosteroid; LABA, long-acting β_2_-agonist; LAMA, long-acting muscarinic antagonist; Sig., significance; Std. error, standard error.

Coefficients from negative binomial generalized linear model, with log (exposure) as offset term. All regions are relative to the USA as the reference group. Significance of sequential inclusion in model from ANOVA: ^***^*p* < 0.001; ^**^*p* < 0.01; ^*^ *p* < 0.05; ^†^*p* < 0.10; ^#^*p* < 0.20.

Table S2. Simplified model coefficients.

|  | Estimate | Std. error | *z*-value | *p*-value | Sig. |
| --- | --- | --- | --- | --- | --- |
| *For all patients* | | | | | |
| (Intercept) | –1.787 | 0.183 | –9.79 | <0.001 | ^***^ |
| No. of exacerbations in prior year | 0.428 | 0.022 | 19.12 | <0.001 | ^***^ |
| log(Eosinophils) | 0.187 | 0.030 | 6.29 | <0.001 | ^***^ |
| Prior ICS use | 0.540 | 0.049 | 10.99 | <0.001 | ^***^ |
| Treated with BD 160 µg | 0.860 | 0.332 | 2.60 | 0.009 | ^**^ |
| Treated with BD 320 µg | 1.204 | 0.317 | 3.80 | <0.001 | ^***^ |
| Treated with GP | –0.209 | 0.118 | –1.77 | 0.077 | ^†^ |
| Treated with FF | 0.000 | 0.106 | –0.003 | 0.998 |  |
| Treated with GP and FF | 0.159 | 0.130 | 1.23 | 0.219 |  |
| Treated with BD 320 µg and FF | 0.083 | 0.211 | 0.39 | 0.696 |  |
| Treated with BD 160 µg, GP and FF | 0.095 | 0.093 | 1.02 | 0.307 |  |
| Treated with BD 320 µg, GP and FF | 0.007 | 0.070 | 0.10 | 0.921 |  |
| *Interaction terms for those receiving budesonide only* | | | | | |
| BD 160 µg: No. of exacerbations in prior year | –0.096 | 0.040 | –2.37 | 0.018 | ^*^ |
| BD 320 µg: No. of exacerbations in prior year | –0.081 | 0.031 | –2.62 | 0.009 | ^**^ |
| BD 160 µg: log(Eosinophils) | –0.143 | 0.061 | –2.35 | 0.019 | ^*^ |
| BD 320 µg: log(Eosinophils) | –0.221 | 0.046 | –4.75 | <0.001 | ^***^ |
| BD 160 µg: Prior ICS use | –0.348 | 0.093 | –3.75 | <0.001 | ^***^ |
| BD 320 µg: Prior ICS use | –0.322 | 0.073 | –4.43 | <0.001 | ^***^ |

ANOVA, analysis of variance; BD, budesonide; FF, formoterol fumarate; GP, glycopyrrolate; ICS, inhaled corticosteroid.

Coefficients from negative binomial generalized linear model, with log (exposure) as offset term. Significance of sequential inclusion in model from ANOVA: ****p* < 0.001; ** *p* < 0.01; * *p* < 0.05; ^†^*p* < 0.10.

Figure S1. Variable importance (prognostic value).


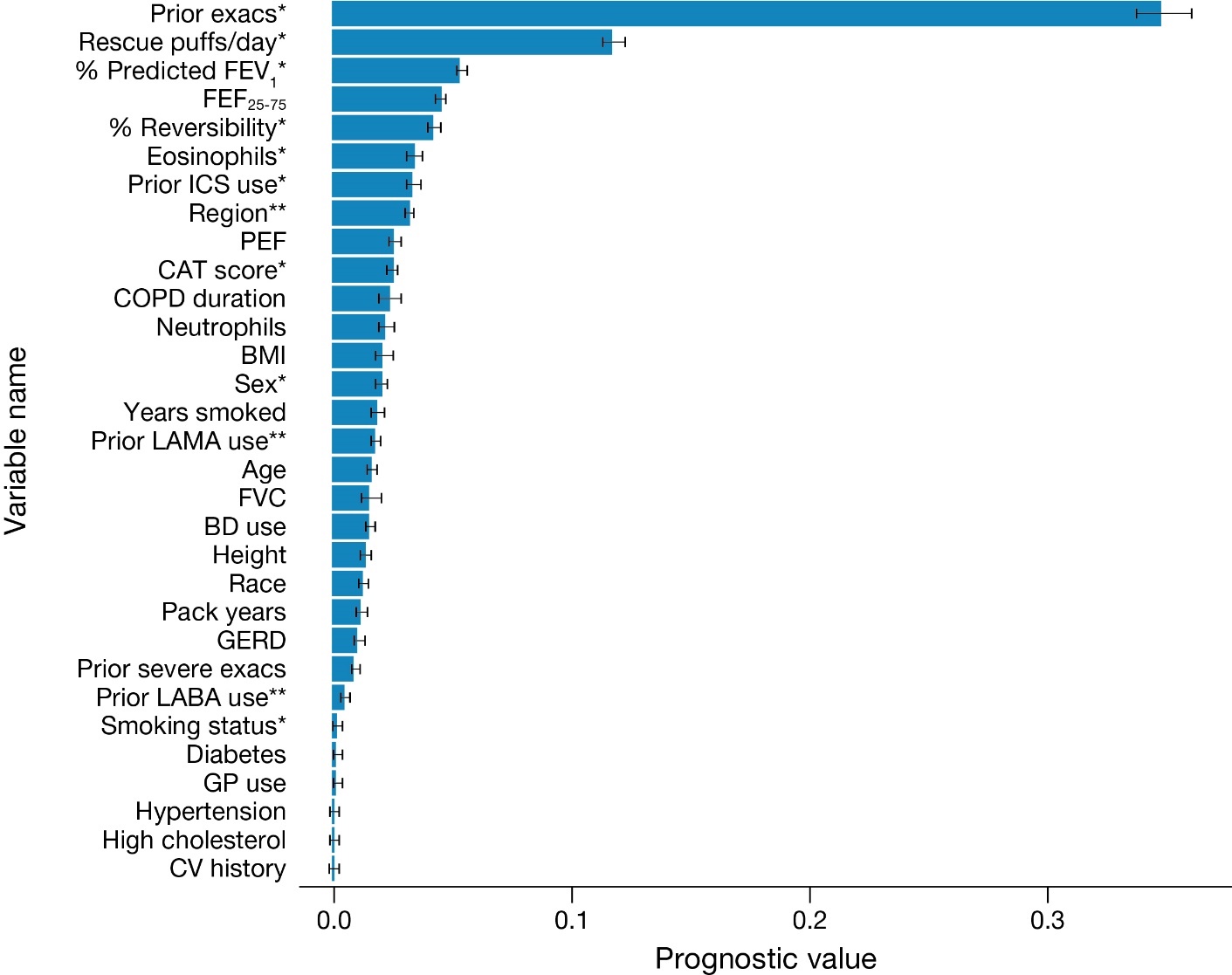


BD, budesonide; BMI, body mass index; CAT, COPD Assessment Test; COPD, chronic obstructive pulmonary disease; CV, cardiovascular; exacs, exacerbations; FEF_25–75_, forced expiratory flow at 25–75% of FVC; FEV_1_, forced expiratory volume in 1 second; FVC, forced vital capacity; GERD, gastroesophageal reflux disease; GP, general practitioner; ICS, inhaled corticosteroid; PEF, peak expiratory flow; LABA, long-acting β_2_-agonist; LAMA, long-acting muscarinic antagonist.

Relative variable importance from gradient boosting machine learning algorithm.

^*^Expected model covariates confirmed as important.

^**^Additional covariates identified and added to the model. Further spirometry parameters were identified but not added due to collinearity.

Figure S2. Rootogram of predicted *versus* actual distribution in (A) training set and (B) test set.


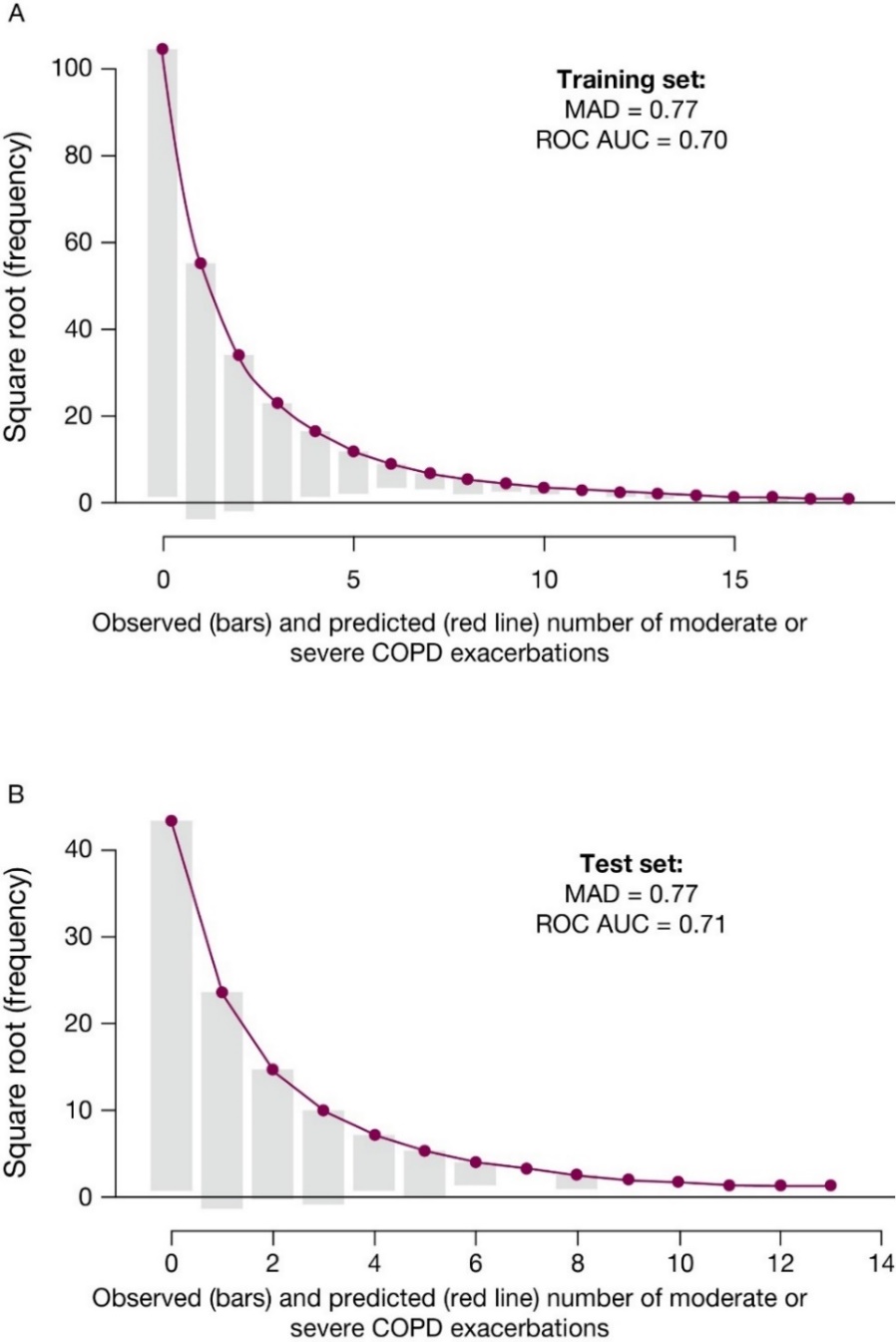


AUC, area under the ROC curve; COPD, chronic obstructive pulmonary disease; MAD, median absolute difference between predicted and observed rate; ROC, receiver operating characteristic.

Bars indicate the observed frequencies and the line represents predicted frequencies. Departures from expected frequencies and any pattern in those departures may be observed against the reference line at y = 0. Square roots are provided rather than untransformed values so that deviations can be visualized across a large range of frequencies, rather than bars being small for frequent exacerbators. Frequencies are lower in the test set (*N* = 2880) compared with the training set (*N* = 16,314) as they represent the square root of the patient number, i.e. a frequency of 20 is equivalent to 400 patients.
